# An ortho­rhom­bic polymorph of pyrazino­[2,3-*f*][1,10]phenanthroline-2,3-dicarbonitrile

**DOI:** 10.1107/S1600536811047039

**Published:** 2011-11-09

**Authors:** Wei Yang, Qi-Ming Qiu, Li-Li Zhou, Qiong-Hua Jin, Cun-Lin Zhang

**Affiliations:** a Department of Chemistry, Capital Normal University, Beijing 100048, People’s Republic of China; b Research Center for Import–Export Chemicals Safety of the General Administration of Quality Supervision, Inspection and Quarantine of the People’s Republic of China (AQSIQ), Chinese Academy of Inspection and Quarantine, Beijing 100123, People’s Republic of China; c Key Laboratory of Terahertz Optoelectronics, Ministry of Education, Department of Physics, Capital Normal University, Beijing 100048, People’s Republic of China

## Abstract

The title compound, C_16_H_6_N_6_, is a polymorph of the previously reported structure [Kozlov & Goldberg (2008 ▶). *Acta Cryst*. C**64**, o498–o501]. Unlike the previously reported monoclinic polymorph (space group *P*2_1_/*c*, *Z* = 8), the title compound reveals ortho­rhom­bic symmetry (space group *Pnma*, *Z* = 4). The mol­ecule shows crystallographic mirror symmetry, while the previously reported structure exhibits two independent mol­ecules per asymmetric unit. In the title compound, adjacent mol­ecules are essentially parallel along the *c* axis and tend to be vertical along the *b* axis with dihedral angles of 72.02 (6)°. However, in the reported polymorph, the entire crystal structure shows an anti­parallel arrangement of adjacent columns related by inversion centers and the two independent mol­ecules are nearly parallel with a dihedral angle of 2.48 (6)°.

## Related literature

For ligands based on 1,10-phenanthroline in coordination chemistry, see: Rabaca *et al.* (2008 ▶); Stephenson *et al.* (2008 ▶). For reports of the title compound in coordination chemistry, see: Kulkarni *et al.* (2004 ▶); Stephenson & Hardie (2006 ▶); Xiao *et al.* (2011 ▶); Xu *et al.* (2002 ▶). For examples of polymorphism, see: Demirtaş *et al.* (2011 ▶); Jiang *et al.* (2000 ▶); Okabe *et al.* (2001 ▶); Pan & Chen (2009 ▶); Ramos Silva *et al.* (2011 ▶); Thallapally *et al.* (2004 ▶). For the previously reported polymorph, see: Kozlov & Goldberg (2008 ▶). For related structures, see: Kozlov *et al.* (2008 ▶).
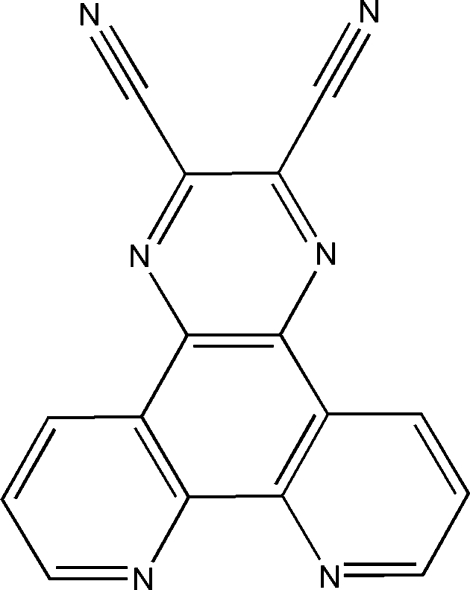


## Experimental

### 

#### Crystal data

C_16_H_6_N_6_
*M* *_r_* = 282.27
Orthorhombic, 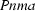

*a* = 14.1055 (13) Å
*b* = 16.3331 (14) Å
*c* = 5.2694 (4) Å
*V* = 1214.00 (18) Å^3^
*Z* = 4
Mo *K*α radiation
μ = 0.10 mm^−1^
*T* = 293 K
0.45 × 0.30 × 0.26 mm

#### Data collection

Bruker SMART CCD area-detector diffractometer
Absorption correction: multi-scan (*SADABS*; Bruker, 2007 ▶) *T*_min_ = 0.956, *T*_max_ = 0.974
4543 measured reflections
1113 independent reflections
759 reflections with *I* > 2σ(*I*)
*R*_int_ = 0.046

#### Refinement

*R*[*F* ^2^ > 2σ(*F* ^2^)] = 0.041
*wR*(*F* ^2^) = 0.119
*S* = 1.06
1113 reflections
100 parameters
H-atom parameters constrained
Δρ_max_ = 0.20 e Å^−3^
Δρ_min_ = −0.24 e Å^−3^

### 

Data collection: *SMART* (Bruker, 2007 ▶); cell refinement: *SAINT-Plus* (Bruker, 2007 ▶); data reduction: *SAINT-Plus*; program(s) used to solve structure: *SHELXS97* (Sheldrick, 2008 ▶); program(s) used to refine structure: *SHELXL97* (Sheldrick, 2008 ▶); molecular graphics: *SHELXTL* (Sheldrick, 2008 ▶) and *ORTEPIII* (Burnett & Johnson, 1996 ▶); software used to prepare material for publication: *SHELXTL*.

## Supplementary Material

Crystal structure: contains datablock(s) global, I. DOI: 10.1107/S1600536811047039/zq2127sup1.cif

Structure factors: contains datablock(s) I. DOI: 10.1107/S1600536811047039/zq2127Isup2.hkl

Supplementary material file. DOI: 10.1107/S1600536811047039/zq2127Isup3.cml

Additional supplementary materials:  crystallographic information; 3D view; checkCIF report

## Figures and Tables

**Fig. 1.**
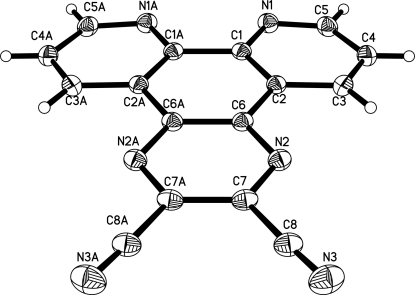
Perspective view of a basic unit of the title complex. Atoms are displayed as elliposoids at the 50% probability level at ca 293 K.

**Fig. 2.**
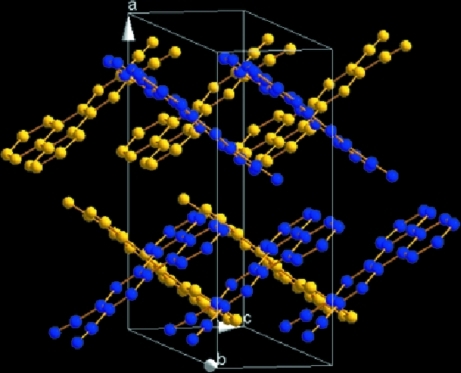
The crystal packing of the title complex.

## supplementary crystallographic information

### Comment

Ligands based on 1,10-phenanthroline are attractive building blocks for the formation of metal-organic complexes bearing diverse dimensionalities (Rabaca *et al.*, 2008; Stephenson *et al.*, 2008). Among 1,10-phenanthroline derivatives, the DICNQ (DICNQ = pyrazino[2,3-*f*][1,10]phenanthroline-2,3-dicarbonitrile) (Xu *et al.*, 2002; Kulkarni *et al.*, 2004) has a big conjugated system and multiple coordination including strong chelating phenanthroline ring, bridging pyrazine ring and cyanite groups, so it is good in synthesizing luminescent complexes containing multifunctional ligands. Up to date only a few complexes of DICNQ are reported, such as [Co(dicnq)_2_Br_2_] and [Cu(dicnq)_2_Br_2_][Cu(dicnq)_2_Br]Br.(CH_3_CN).2(H_2_O) (Stephenson & Hardie, 2006). Very recently, we synthesized five Cu(I) complexes [Cu(PPh_3_)(C_16_H_6_N_6_)*X*] (C_16_H_6_N_6 _= pyrazino[2,3-*f*][1,10]phenanthroline-2,3-dicarbonitrile; *X* = Cl, Br, I, CN, SCN) (Xiao *et al.*, 2011). During our attempts to synthesize Cu(II) complexes of DICNQ and 4,4'-bipyridine, we obtained crystals of a new orthorhombic polymorph of the solvent-free DICNQ.

Polymorphism can potentially be found in any crystalline material including polymers, minerals, and metals, and is related to allotropy, which refers to elemental solids. When polymorphism exists as a result of difference in crystal packing, it is called packing polymorphism. The different crystal types probably are the result of hydration, solvation or the effect of synthesis methods. There are recently reported examples of organic polymorphs: 2-(pyrimidin-2-ylsulfanyl) acetic acid, which is able to form monoclinic and orthorhombic crystals (Ramos Silva *et al.*, 2011; Pan & Chen, 2009). 3,4,5-Trihydroxybenzoic acid monohydrate is found to form three polymorphs (Demirtaş *et al.*, 2011; Jiang *et al.*, 2000; Okabe *et al.*, 2001). We herein report a new orthorhombic polymorph of DICNQ (1).

*ORTEPIII* (Burnett & Johnson, 1996) representation of the title compound (1) is shown in Fig. 1. The title compound belongs to orthorhombic system, space group *Pnma*, consisting of one symmetric unit of single molecule, unlike the reported monoclinic polymorph (space group *P*2_1_/*c*), (2), (Kozlov & Goldberg, 2008), which consists of two crystallographically independent molecules.

The phenanthroline fragment of both polymorphs is nearly planar, and a similar bending of the cyano groups from the plane of the phenanthroline residues can be observed. The deviating distance of the cyano groups from the plane in (1) (0.182 (7) Å) is obviously bigger than the corresponding deviating distances in (2) (0.0345 Å and 0.0374 Å).

The packing pattern differs from the reported polymorph. In the title compound, there are two packing arrangements (Fig. 2), adjacent molecules are essentially parallel along the *c* axis and tend to be vertical along the *b* axis with dihedral angles of 72.02 (6)°. Howerver, in the reported polymorph the entire crystal structure shows an antiparallel arrangement of the adjacent columns related by inversion centers, and the two independent molecules are nearly parallel with a dihedral angle of 2.48 (6)°.

In addition, the intermolecular assembly of (1) is dominated by π-π stacking of overlaps among phenanthroline fragments and π-π stacking between phenanthroline fragment and cyano group of adjacent molecules (Fig. 2), with an interplanar distance between the parallel phenanthroline rings in the two adjacent molecules of 3.030 (1) Å. In contrast, in (2) and the ethanol solvate of DICNQ (C_16_H_6_N_6_).(C_2_H_6_O) (3) (Kozlov *et al.*, 2008), adjacent molecules overlap only through their phenanthroline fragments with the shortest interplanar distance of 3.380 (3) Å for (2), 3.27 Å and 3.40 Å for (3).

Various methods and conditions of the crystallization process are the main reason responsible for the formation of different polymorphic forms (Thallapally *et al.*, 2004). The two polymorphs (1) and (2) are prepared under different catalysts. (1) was prepared under the catalysis of CuCl_2_.2H_2_O and 4,4'-bipy, while (2) was prepared under the catalysis of [Pd(PPh_3_)Cl_2_].

### Experimental

A mixture of CuCl_2_.2H_2_O (0.0178 g, 0.1 mmol) in 5 ml CH_2_Cl_2_ and DICNQ (0.0290 g, 0.1 mmol) in 3 ml CH_3_CN was stirred for 30 min, then a solution of 4,4'-bipyridine (0.0202 g, 0.1 mmol) in 2 ml CH_3_CN was added under continuous stirring. The resulting solution was refluxed for 5 h and filtered. The filtrate was allowed to evaporate slowly at room temperature to yield yellow block crystals of the title complex. Crystals suitable for single-crystal X-ray diffraction were selected directly from the sample as prepared.

IR (KBr, disc: v/cm^-1^): 3466.82 s, 1587.57*m*, 1571.17*m*, 1505.59*m*, 1459.84*m*, 1444.23*m*, 1384.77*m*, 1372.26*m*, 1331.97w, 1264.07w, 1219.29*m*, 1141.93*m*, 1123.84w, 1073.56w, 1028.64w, 975.09w, 817.56*m*, 743.23*m*, 688.54w, 618.37w, 527.98w, 441.00w, 420.02w, 409.42w.

### Refinement

H atoms were positioned geometrically and refined using a riding model with C—H = 0.93 Å and with *U*_iso_(H) = 1.2*U*_eq_(C).

### Crystal data

**Table:**

C_16_H_6_N_6_	*F*(000) = 576
*M**_r_* = 282.27	*D*_x_ = 1.544 Mg m^−^^3^
Orthorhombic, *P**n**m**a*	Mo *K*α radiation, λ = 0.71073 Å
Hall symbol: -P 2ac 2n	Cell parameters from 1184 reflections
*a* = 14.1055 (13) Å	θ = 2.9–27.5°
*b* = 16.3331 (14) Å	µ = 0.10 mm^−^^1^
*c* = 5.2694 (4) Å	*T* = 293 K
*V* = 1214.00 (18) Å^3^	Block, yellow
*Z* = 4	0.45 × 0.30 × 0.26 mm

### Data collection

**Table:**

Bruker SMART CCD area-detector diffractometer	1113 independent reflections
Radiation source: fine-focus sealed tube	759 reflections with *I* > 2σ(*I*)
graphite	*R*_int_ = 0.046
phi and ω scans	θ_max_ = 25.0°, θ_min_ = 2.9°
Absorption correction: multi-scan (*SADABS*; Bruker, 2007)	*h* = −16→16
*T*_min_ = 0.956, *T*_max_ = 0.974	*k* = −19→7
4543 measured reflections	*l* = −6→6

### Refinement

**Table:**

Refinement on *F*^2^	Primary atom site location: structure-invariant direct methods
Least-squares matrix: full	Secondary atom site location: difference Fourier map
*R*[*F*^2^ > 2σ(*F*^2^)] = 0.041	Hydrogen site location: inferred from neighbouring sites
*wR*(*F*^2^) = 0.119	H-atom parameters constrained
*S* = 1.06	*w* = 1/[σ^2^(*F*_o_^2^) + (0.0556*P*)^2^ + 0.3018*P*] where *P* = (*F*_o_^2^ + 2*F*_c_^2^)/3
1113 reflections	(Δ/σ)_max_ < 0.001
100 parameters	Δρ_max_ = 0.20 e Å^−^^3^
0 restraints	Δρ_min_ = −0.24 e Å^−^^3^

### Special details

**Table:**

Geometry. All e.s.d.'s (except the e.s.d. in the dihedral angle between two l.s. planes) are estimated using the full covariance matrix. The cell e.s.d.'s are taken into account individually in the estimation of e.s.d.'s in distances, angles and torsion angles; correlations between e.s.d.'s in cell parameters are only used when they are defined by crystal symmetry. An approximate (isotropic) treatment of cell e.s.d.'s is used for estimating e.s.d.'s involving l.s. planes.
Refinement. Refinement of *F*^2^ against ALL reflections. The weighted *R*-factor *wR* and goodness of fit *S* are based on *F*^2^, conventional *R*-factors *R* are based on *F*, with *F* set to zero for negative *F*^2^. The threshold expression of *F*^2^ > σ(*F*^2^) is used only for calculating *R*-factors(gt) *etc*. and is not relevant to the choice of reflections for refinement. *R*-factors based on *F*^2^ are statistically about twice as large as those based on *F*, and *R*- factors based on ALL data will be even larger.

### Fractional atomic coordinates and isotropic or equivalent isotropic displacement parameters (Å^2^)

**Table:**

	*x*	*y*	*z*	*U*_iso_*/*U*_eq_	
N1	0.56932 (11)	0.33317 (10)	−0.1078 (3)	0.0347 (5)	
N2	0.77953 (11)	0.33585 (9)	0.6140 (3)	0.0322 (4)	
N3	0.92249 (13)	0.37680 (13)	1.1146 (4)	0.0539 (6)	
C1	0.61818 (12)	0.29508 (11)	0.0786 (3)	0.0279 (5)	
C2	0.67134 (12)	0.33802 (11)	0.2603 (3)	0.0281 (5)	
C3	0.67273 (13)	0.42373 (12)	0.2476 (4)	0.0349 (5)	
H3	0.7060	0.4542	0.3673	0.042*	
C4	0.62440 (14)	0.46176 (13)	0.0564 (4)	0.0381 (6)	
H4	0.6249	0.5185	0.0422	0.046*	
C5	0.57451 (13)	0.41428 (12)	−0.1161 (4)	0.0369 (5)	
H5	0.5426	0.4410	−0.2463	0.044*	
C6	0.72648 (12)	0.29328 (11)	0.4470 (3)	0.0285 (5)	
C7	0.83106 (12)	0.29288 (12)	0.7773 (4)	0.0317 (5)	
C8	0.88437 (14)	0.33918 (13)	0.9640 (4)	0.0372 (5)	

### Atomic displacement parameters (Å^2^)

**Table:**

	*U*^11^	*U*^22^	*U*^33^	*U*^12^	*U*^13^	*U*^23^
N1	0.0333 (9)	0.0368 (10)	0.0339 (10)	0.0021 (7)	−0.0025 (8)	0.0040 (8)
N2	0.0305 (9)	0.0357 (9)	0.0303 (9)	−0.0020 (7)	0.0014 (8)	−0.0007 (8)
N3	0.0476 (11)	0.0703 (14)	0.0437 (12)	−0.0084 (10)	−0.0041 (10)	−0.0142 (11)
C1	0.0249 (9)	0.0318 (10)	0.0271 (11)	0.0011 (8)	0.0041 (8)	0.0010 (9)
C2	0.0255 (9)	0.0291 (10)	0.0296 (11)	0.0002 (8)	0.0036 (8)	0.0018 (9)
C3	0.0335 (11)	0.0312 (10)	0.0401 (13)	−0.0031 (9)	−0.0007 (10)	−0.0031 (10)
C4	0.0353 (11)	0.0302 (10)	0.0488 (14)	0.0018 (9)	0.0026 (11)	0.0059 (10)
C5	0.0354 (11)	0.0370 (12)	0.0382 (12)	0.0052 (9)	−0.0005 (10)	0.0082 (10)
C6	0.0253 (9)	0.0319 (9)	0.0283 (10)	−0.0016 (8)	0.0028 (8)	−0.0029 (8)
C7	0.0264 (10)	0.0428 (11)	0.0261 (11)	−0.0027 (8)	0.0008 (9)	−0.0014 (9)
C8	0.0314 (11)	0.0480 (13)	0.0321 (12)	−0.0018 (9)	0.0013 (10)	−0.0013 (11)

### Geometric parameters (Å, °)

**Table:**

N1—C5	1.327 (3)	C3—C4	1.366 (3)
N1—C1	1.352 (2)	C3—H3	0.9300
N2—C7	1.327 (2)	C4—C5	1.387 (3)
N2—C6	1.348 (2)	C4—H4	0.9300
N3—C8	1.138 (3)	C5—H5	0.9300
C1—C2	1.404 (2)	C6—C6^i^	1.414 (4)
C1—C1^i^	1.473 (4)	C7—C7^i^	1.401 (4)
C2—C3	1.402 (3)	C7—C8	1.451 (3)
C2—C6	1.451 (2)		
			
C5—N1—C1	117.07 (17)	C3—C4—H4	120.6
C7—N2—C6	117.02 (16)	C5—C4—H4	120.6
N1—C1—C2	122.55 (17)	N1—C5—C4	124.37 (19)
N1—C1—C1^i^	117.41 (10)	N1—C5—H5	117.8
C2—C1—C1^i^	119.97 (11)	C4—C5—H5	117.8
C3—C2—C1	118.29 (17)	N2—C6—C6^i^	121.05 (10)
C3—C2—C6	121.85 (17)	N2—C6—C2	118.69 (16)
C1—C2—C6	119.80 (17)	C6^i^—C6—C2	120.23 (10)
C4—C3—C2	118.85 (18)	N2—C7—C7^i^	121.93 (10)
C4—C3—H3	120.6	N2—C7—C8	116.61 (17)
C2—C3—H3	120.6	C7^i^—C7—C8	121.41 (11)
C3—C4—C5	118.85 (18)	N3—C8—C7	177.0 (2)
			
C5—N1—C1—C2	1.0 (3)	C3—C4—C5—N1	0.7 (3)
C5—N1—C1—C1^i^	−175.87 (13)	C7—N2—C6—C6^i^	−0.18 (19)
N1—C1—C2—C3	0.5 (3)	C7—N2—C6—C2	−178.16 (15)
C1^i^—C1—C2—C3	177.37 (12)	C3—C2—C6—N2	0.7 (3)
N1—C1—C2—C6	−176.57 (15)	C1—C2—C6—N2	177.73 (15)
C1^i^—C1—C2—C6	0.26 (19)	C3—C2—C6—C6^i^	−177.26 (13)
C1—C2—C3—C4	−1.5 (3)	C1—C2—C6—C6^i^	−0.26 (19)
C6—C2—C3—C4	175.52 (17)	C6—N2—C7—C7^i^	0.18 (19)
C2—C3—C4—C5	1.0 (3)	C6—N2—C7—C8	−177.14 (15)
C1—N1—C5—C4	−1.7 (3)		

Symmetry codes: (i) *x*, −*y*+1/2, *z*.
